# Computer-aided drug design combined network pharmacology to explore anti-SARS-CoV-2 or anti-inflammatory targets and mechanisms of Qingfei Paidu Decoction for COVID-19

**DOI:** 10.3389/fimmu.2022.1015271

**Published:** 2022-12-23

**Authors:** Zixuan Wang, Jiuyu Zhan, Hongwei Gao

**Affiliations:** School of Life Science, Ludong University, Yantai, Shandong, China

**Keywords:** COVID-19, herb, target, active component, anti-inflammatory, anti-SARS-CoV-2

## Abstract

**Introduction:**

Coronavirus Disease-2019 (COVID-19) is an infectious disease caused by SARS-CoV-2. Severe cases of COVID-19 are characterized by an intense inflammatory process that may ultimately lead to organ failure and patient death. Qingfei Paidu Decoction (QFPD), a traditional Chines e medicine (TCM) formula, is widely used in China as anti-SARS-CoV-2 and anti-inflammatory. However, the potential targets and mechanisms for QFPD to exert anti-SARS-CoV-2 or anti-inflammatory effects remain unclear.

**Methods:**

In this study, Computer-Aided Drug Design was performed to identify the antiviral or anti-inflammatory components in QFPD and their targets using Discovery Studio 2020 software. We then investigated the mechanisms associated with QFPD for treating COVID-19 with the help of multiple network pharmacology approaches.

**Results and discussion:**

By overlapping the targets of QFPD and COVID-19, we discovered 8 common targets (RBP4, IL1RN, TTR, FYN, SFTPD, TP53, SRPK1, and AKT1) of 62 active components in QFPD. These may represent potential targets for QFPD to exert anti-SARS-CoV-2 or anti-inflammatory effects. The result showed that QFPD might have therapeutic effects on COVID-19 by regulating viral infection, immune and inflammation-related pathways. Our work will promote the development of new drugs for COVID-19.

## 1 Introduction

The pandemic of Coronavirus Disease-2019 (COVID-19) has posed an unprecedented crisis to global public health (1–3). The main symptoms of COVID-19 infection are fever, coughing, shortness of breathing and also death (4). Traditional Chinese medicine (TCM) formulas was widely used in China against COVID-19, especially in 2020, in the absence of specific drugs and vaccines (5–7). Based on the current clinical investigation, the treatment of inflammatory storms has been proposed as a critical part of rescuing severe COVID-19 (8–11). Qingfei Paidu Decoction (QFPD) has become one of the most used compounds due to its potential role in the treatment of COVID-19 (12–14). QFPD is composed of 20 herbs (15), namely *Agaric* (Zhuling), *Oriental waterplantain tuber* (Zexie), *Largehead atractylodes rhizome* (Baizhu), *Cassia twig* (Guizhi), *Chinese ephedra herb* (Mahuang), *Semen armeniacae amarum* (Xingren), *Poria cocos* (Fuling), *Chinese thorowax root* (Chaihu), *Baikal skullcap root* (Huangqin), *Pinellinae rhizoma praeparatum* (Jiangbanxia), *Common ginger rhizome* (Shengjiang), *Tatarian aster root and rhizomes* (Ziwan), *Common coltsfoot flower* (Kuandonghua), *Blackberrglily rhizome* (Shegan), *Manchurian wildginger herb* (Xixin), *Common yam rhizome* (Shanyao), *Immature bitter orange* (Zhishi), *Wrinkled gianthyssop herb* (Huoxiang), *Dried tangerine peel* (Chenpi), and *Baked radix glycyrrhizae* (Zhigancao). The combination of these herbs is used to reduce mortality and improve cure rates in patients with COVID-19 (16).

Computer-Aided Drug Design (CADD) is a method for developing lead compounds by theoretical calculation, simulation, and prediction of the relationship between drugs and receptors (17). Network pharmacology is a powerful tool to reflect the pharmacological effects and mechanisms of TCM (18–20). The concept of holism for TCM has much in common with the major points of network pharmacology, in which the general “one target, one drug” mode is shifted to a new “multi-target, multi-component” mode (21). The research method of CADD combined with network pharmacology can be used to reveal the mystery of the “multi-target, multi-component and multi-path” of TCM formulas. This method greatly improves the success rate of drug research and saves the cost of drug development.

QFPD has the potential therapeutic effects for COVID-19 intervention in China, but how to take advantage of its anti-SARS-CoV-2 and anti-inflammatory effects deserves further exploration. Therefore, we aim to screen the antiviral or anti-inflammatory components in QFPD and their targets using Discovery Studio 2020 (DS2020) software. We compared the targets regulated by the active components in QFPD with the COVID-19 targets recorded in the Genecards database (https://www.genecards.org) and obtained their common targets. With the help of network pharmacology, we investigated how QFPD regulates the body from various aspects through multiple targets and multiple pathways. The results provided some vital information for the precise clinical medication and improved the therapeutic ability of TCM for COVID-19.

## 2 Experimental section

### 2.1 Screening the active components in QFPD from the database

Most active components of 20 herbs in QFPD were collected through the Traditional Chinese Medicines Database (TCMdb). It was a new tool with 23033 active components to support the modernization of TCM (22–24). In addition, the Traditional Chinese Medicine Systems Pharmacology Database (TCMSP database; http://tcmspw.com/tcmsp.php) was used to supplement active components. The TCMSP database integrated active components, relevant diseases, targets, and pharmacokinetic data of drugs, providing a new platform for studying the mechanism of drug action systematically (25). Only components with antiviral or anti-inflammatory effects were retained for later studies.

### 2.2 Ligand preparation and the prediction of absorption, distribution, metabolism, excretion, and toxicity properties

The Prepare Ligands protocol helped to prepare ligands for input components, performing tasks such as removing duplicates, enumerating isomers and tautomers. This study performed the following steps to complete this operation: changing ionization, generating tautomers, generating isomers, and fixing bad valencies. Then, the ligands of active components needed to be minimized in batches by Minimize Ligands protocol before ADMET properties’ prediction. ADMET refers to the Absorption, Distribution, Metabolism, Excretion, and Toxicity properties of a molecule within an organism (26, 27). The ADMET properties predicted in this study were hepatotoxicity, Blood-brain barrier penetration (BBB) and Human intestinal absorption (HIA) (28, 29). Optimizing these two properties during early drug discovery was crucial for reducing ADMET problems later in development.

#### 2.3 The prediction of toxicological properties and druggability screening

Toxicity Prediction by Komputer Assisted Technology (TOPKAT) accurately and rapidly assessed the toxicity of chemicals based solely on their 2D molecular structure. It could assess the toxicological properties of candidate active components with a range of robust, cross-validated, and Quantitative Structure-Toxicity Relationship (QSTR) models (30). The toxicological properties we predicted in this study were the Ames test, Rodent Carcinogenicity, Aerobic Biodegradability, and oral LD50 in rats. After the predicted results were obtained, all active components that exceed these four properties’ optimal prediction space (OPS) needed to be deleted manually. In order to exclude active components with poor absorption, permeation, and oral bioavailability, it was necessary to ensure that the screened active components comply with Lipinski’s rule of five (31) and Veber’s rules (32, 33). The active components that did not meet these rules will be automatically deleted at the end of the calculation. The reserved active components had better pharmacokinetic properties and higher bioavailability in the metabolism of organisms.

### 2.4 Performing reverse finding target

The technique of reverse finding target was the core of this study. Reverse finding target process was to match the pharmacophore models with active components which had high credibility after a series of screening. In addition, the matching degree of pharmacophore models and active components can be distinguished by different colors in the “Heat map of Ligand profiler”. Generally, the higher the matching between the pharmacophore models and the active components, the higher the confidence of the targets corresponding to the pharmacophore models. Based on these pharmacophore models, we can identify the target proteins regulated by the active components of QFPD. We then used the search function in the Protein Data Bank (PDB) database to convert the target protein names to standard gene names. COVID-19 targets recorded in the Genecards database and QFPD targets regulated by active ingredients in QFPD were compared to get common targets. These common targets represented potential targets for QFPD to exert anti-SARS-CoV-2 or anti-inflammatory effects.

### 2.5 Construction of protein-protein interaction network

The interaction between the targets was illustrated as a PPI network. The construction of the PPI network was realized *via* the Search Tool for the Retrieval of Interacting Genes (STRING; https://string-db.org/). It gathered a large number of information resources, mainly for storing PPI data identified by experiments, calculating predicted data, and collecting public text (34).

### 2.6 Construction of “herb-active component-target” interaction network diagram

The “herb-active component-target” interaction network was plotted by the major constituents of QFPD and their targets using Cytoscape 3.8.0. The network constructed by this information was represented as nodes and edges with related data attributes, which revealed the close relationship between diseases, targets, and drugs, and provided ideas for further study of multi-target and multi-component TCM formula.

### 2.7 Pathway analysis of QFPD

Gene set enrichment analysis (GSEA) was performed on transcriptome sequencing data of COVID-19 from Gene Expression Omnibus (GEO) database using GSEA 4.1.0. The COVID-19 group consisted of 30 samples, which were organized into gene expression matrix. Based on the expression of the target, they were divided into target high expression and low expression groups for GSEA analysis.

The potential targets of QFPD were submitted to DAVID (https://david.ncifcrf.gov/) to analyze Gene Ontology (GO) function enrichment and Kyoto Encyclopedia of Genes and Genomes (KEGG) pathway enrichment. GO analysis was involved in terms of cellular component (CC), molecular function (MF), as well as biological process (BP) (35). CC was defined as the active sites of gene products in cells. MF was considered as the biochemical activity. BP involved the contribution of genes or genetic products to biological objectives. KEGG was a highly integrated database for biological interpretation of wholly sequenced genomes through pathway mapping (36).

## 3 Results and discussion

### 3.1 Screening the active components in QFPD from the database

A total of 108 active components in QFPD were selected from TCMdb and TCMSP database based on the above criteria about antiviral or anti-inflammatory effects. The basic information of 108 active components was showed in **Table 1**.

**Table 1:** Active components in QFPD.

Herb	Active components	Effect	References
Huangqin	Isoscutellarein	antiviral	(37, 38)
	Baicalein	anti-inflammatory	
	Baicalin	anti-inflammatory	
	Eriodictyol	anti-inflammatory	
	Oroxylin A	anti-inflammatory	
	beta-Sitosterol	anti-inflammatory	
	Wogonin	anti-inflammatory	
	Wogonoside	anti-inflammatory	
	Chrysin	anti-inflammatory, antiviral	
Jiangbanxia	beta-Sitosterol	anti-inflammatory	(39)
Kuandonghua	Gallic acid	anti-inflammatory	(40–42)
	Hyperin	anti-inflammatory	
	Rutin	anti-inflammatory, antiviral	
Shegan	Tectoridin	anti-inflammatory	(43, 44)
	Tectorigenin	anti-inflammatory	
	Mangiferin	anti-inflammatory, antiviral	
Xixin	Sesamin	antiviral	(45–47)
	Aristolochic acid	antiviral	
	(+)-Eudesma-4(15),7(11)-dien-8-one	anti-inflammatory	
	Terpinen-4-ol	anti-inflammatory	
Shanyao	beta-Sitosterol	anti-inflammatory	(39)
Zhishi	Tangeretin	antiviral	(48, 49)
	5,7,4'-Trimethoxyflavone	antiviral	
	Hesperidin	antiviral	
	Apigenin-7-O-neohesperidoside	antiviral	
	Lonicerin	anti-inflammatory	
	Naringin	anti-inflammatory, antiviral	
Chenpi	Hesperidin	antiviral	(49, 50)
	Naringin	anti-inflammatory, antiviral	
Huoxiang	Linalool	antiviral	(51–53)
	Pachypodol	antiviral	
	Acacetin	anti-inflammatory	
	Friedelan-3-one	anti-inflammatory	
	beta-Pinene	anti-inflammatory	
	beta-Sitosterol	anti-inflammatory	
	Cinnamaldehyde	anti-inflammatory	
Mahuang	Kaempferol	antiviral	(54, 55)
	(4S,5R) Ephedroxane	anti-inflammatory	
	Isoquercitrin	anti-inflammatory	
	N-Methylephedrine	anti-inflammatory	
Shengjiang	Linalool	antiviral	(45, 51, 56)
	6-Dehydrogingerdione	anti-inflammatory	
	10-Dehydrogingerdione	anti-inflammatory	
	Geraniol	anti-inflammatory	
	D-Isoborneol	anti-inflammatory	
	L-Isoborneol	anti-inflammatory	
	Terpinen-4-ol	anti-inflammatory	
Xingren	Linalool	antiviral	(51, 57)
	Dihydroquercetin	anti-inflammatory	
	Eriodictyol	anti-inflammatory	
Ziwan	Quercetin	antiviral	(58, 59)
	Friedelan-3-one	anti-inflammatory	
	Anethole	anti-inflammatory	
Baizhu	Atractylenolide I	anti-inflammatory	(60)
	Atractylone	anti-inflammatory	
	(+)-Eudesma-4(15),7(11)-dien-8-one	anti-inflammatory	
Fuling	Dihydroquercetin	anti-inflammatory	(61, 62)
	Astilbin	anti-inflammatory, antiviral	
Guizhi	2-Methoxycinnamaldehyde	anti-inflammatory	(39, 63)
	beta-Sitosterol	anti-inflammatory	
	Cinnamaldehyde	anti-inflammatory, antiviral	
Zhuling	MAN	anti-inflammatory, antiviral	(64)
	GLA	anti-inflammatory, antiviral	
Zexie	Emodin	anti-inflammatory, antiviral	(65–67)
	HMF	anti-inflammatory, antiviral	
	alpha-D-fructofuranose	anti-inflammatory, antiviral	
	EA-fructofuranoside	anti-inflammatory, antiviral	
	stachyose	anti-inflammatory, antiviral	
	NCA	anti-inflammatory, antiviral	
	1-Monolinolein	anti-inflammatory, antiviral	
	stearic acid	anti-inflammatory, antiviral	
	orientalolf	anti-inflammatory, antiviral	
	Sulfoorientalol C	anti-inflammatory, antiviral	
	raffinose	anti-inflammatory, antiviral	
	(2R,3S,4S,5R)-2-ethoxy-2,5-bis(hydroxymethyl)oxolane-3,4-diol	anti-inflammatory, antiviral	
Zhigancao	3,3'-Dimethylquercetin	antiviral	(11, 39, 41, 68–70)
	Glycycoumarin	antiviral	
	Glepidotin D	antiviral	
	Glycyrrhisoflavone	antiviral	
	Glycyrrhizic acid	antiviral	
	Isolicoflavonol	antiviral	
	Licopyranocoumarin	antiviral	
	6,8-Bis(C-beta-glucosyl)-apigenin	anti-inflammatory	
	Isoliquiritin	anti-inflammatory	
	Isoquercitrin	anti-inflammatory	
	Liquiritic acid	anti-inflammatory	
	Pinocembrin	anti-inflammatory	
	beta-Sitosterol	anti-inflammatory	
	Glycyrrhetinic acid	anti-inflammatory	
	Licochalcone A	anti-inflammatory, antiviral	
	Glycyrrhizic acid	anti-inflammatory, antiviral	
	Rutin	anti-inflammatory, antiviral	
Chaihu	Linalool	antiviral	(51, 56, 71–73)
	Saikosaponin C	antiviral	
	Geraniol	anti-inflammatory	
	Isoquercitrin	anti-inflammatory	
	Kaempferitrin	anti-inflammatory	
	Oroxylin A	anti-inflammatory	
	Propapyriogenin A2	anti-inflammatory	
	Pulegone	anti-inflammatory	
	Saikosaponin B2	anti-inflammatory	
	Scoparone	anti-inflammatory	
	alpha-Spinasterol	anti-inflammatory	
	Wogonin	anti-inflammatory	
	Saikosaponin D	anti-inflammatory, antiviral	
	Saikosaponin A	anti-inflammatory, antiviral	
	(E)-3-(3,4-Dimethoxyphenyl)-2-propen-1-yl (Z)-2-[(Z)-2-methyl-2-butenoyloxymethyl] butenoate	anti-inflammatory	
	1-(3,4,5-Trimethoxyphenyl)-2-propenyl 2-(2-methyl-2Z-butenoyloxymethyl)-2Z-butenoate	anti-inflammatory	

### 3.2 Ligand preparation and prediction of ADMET properties

**Figure 1:**
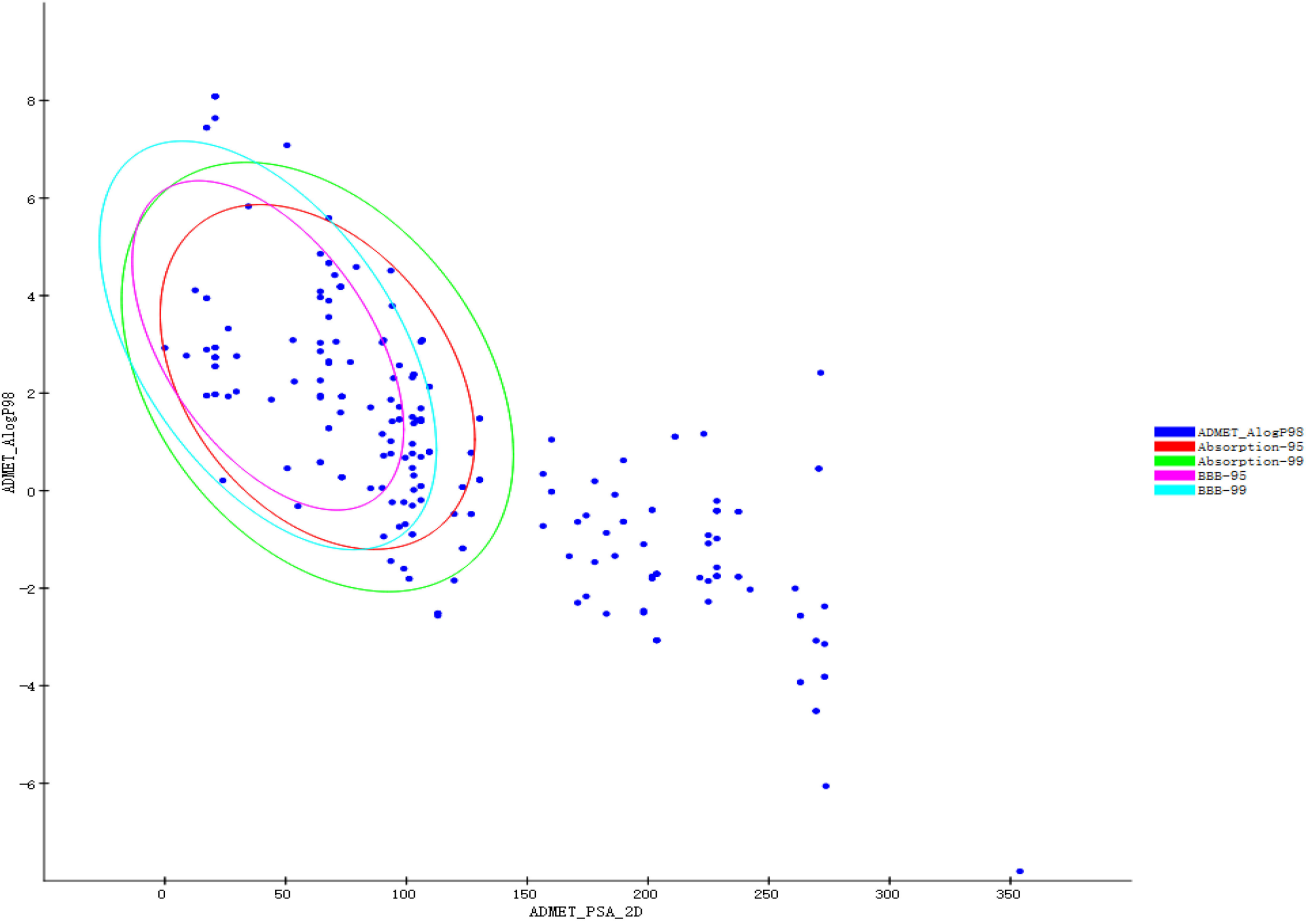
ADMET property prediction results of QFPD.

Prepare Ligands and Minimize Ligands protocols in DS2020 were used to prepare and minimize the structures of 108 active components, respectively. The results showed that 107 tautomers were produced during the preparation process, so the number of active components reached 215. After minimization, the number of active components remained unchanged. Favorable ADMET properties can be considered as essential nature for a candidate drug. As shown in **Figure 1**, the green ellipse represents 99% of the absorption confidence interval, and the blue ellipse represents 99% of the BBB confidence interval. In general, the absorption outside the 99% ellipse tends to drop relatively quickly. If the active component is outside the 99% confidence interval of the BBB model, the prediction of the molecule is considered unreliable. In order to reduce the risk of late-stage attrition, we excluded active components outside the 99% confidence interval of the BBB model and HIA model. In this process, all the active components from the 5 herbs (Jiangbanxia, Chenpi, Fuling, Shanyao, Zhuling) were excluded. In the end, only 97 compounds from 15 herbs were left.

### 3.3 TOPKAT and druggability screening

We checked whether the 97 active components were in the OPS of four toxicological properties (Ames test, Rodent Carcinogenicity, Aerobic Biodegradability, and oral LD50). It can be seen from **Table S1** that the number of candidate active components became 68 after excluding active components that did not meet the OPS. Due to the unsatisfactory results of the active components of Kuandonghua and Ziwan, they should not be further studied. In the process of druggability screening, the program automatically eliminated 2 unqualified active components according to Lipinski’s rule of five and Veber’s rules. Therefore, 66 active components from 13 herbs may become oral drugs.

### 3.4 Reverse finding target

**Figure 2:**
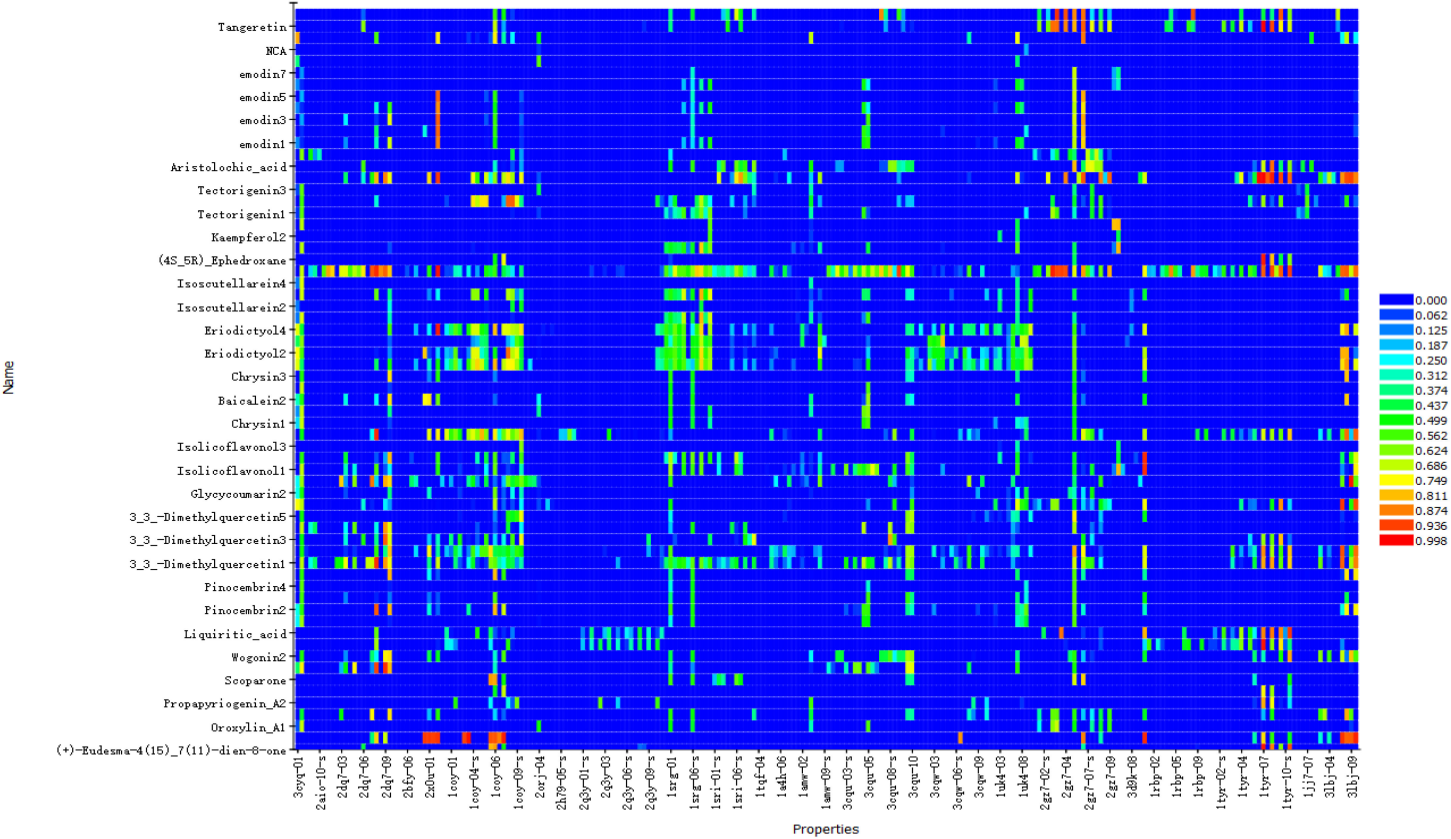
Reverse finding target results of QFPD. The horizontal axis represents the pharmacophore models, and the longitudinal axis represents the active components. Due to the limited size of the picture, we only show more promising pharmacophore models and active components.

The results showed that the corresponding targets of active components in Guizhi did not have antiviral or anti-inflammatory effects. Therefore, 64 active components from 12 herbs were retained in the reverse finding target process. The structures of 64 active components are shown in **Figure S1**. It can be seen from **Figure 2** that the horizontal axis and the longitudinal axis represent the pharmacophore models and the active components, respectively. The color gradient trend is red, yellow, green, and blue on the Heat map. The pharmacophore models with high and low Fit Value are represented by red and blue, respectively. Red means that the matching degree is good. Based on these pharmacophore models, we found that the possible targets of the 64 active components in QFPD were Antigen peptide transporter 1 (TAP1), Retinol-binding protein 4 (RBP4), Interleukin 1 receptor antagonist (IL1RN), Centromere-associated protein E (CENPE), Beta-secretase 1 (BACE1), Transthyretin (TTR), Tyrosine-protein kinase Fyn (FYN), Thyroid hormone receptor alpha (THRA), Pulmonary surfactant-associated protein D (SFTPD), Nuclear receptor subfamily 0 group B member 2 (NR0B2), Cellular tumor antigen p53 (TP53), SRSF protein kinase 1 (SRPK1), RAC-alpha serine/threonine-protein kinase (AKT1) and Protein Mdm4 (MDM4). We compared 14 targets regulated by 64 active components in QFPD with the COVID-19 targets recorded in Genecards databases and found the common targets RBP4 (74), IL1RN (8), TTR (75), FYN (76), SFTPD (77), TP53 (78), SRPK1 (79), and AKT1 (80). The information of 8 potential targets is shown in **Table 2**. FYN, AKT1, SFTPD, SRPK1, and TP53 were SARS-CoV-2 specific targets.

**Table 2:** All pharmacophores and their possible corresponding targets obtained by reverse finding target.

Pharmacophore	Possible target(gene name)	Effect	References
1rbp-02,1rbp-02-s,1rbp-03,1rbp-03-s,1rbp-04,1rbp-04-s,1rbp-05,1rbp-06,1rbp-07-s,1rbp-08,1rbp-08-s,1rbp-09,1rbp-10,1rbp-10-s	1rbp(RBP4)	anti-inflammatory	(74)
1sri-01-s, 1sri-02-s, 1sri-03-s, 1sri-04-s, 1sri-05-s, 1sri-06-s, 1sri-07-s, 1sri-08-s, 1sri-09-s, 1sri-10-s	1sri(IL1RN)	anti-inflammatory	(8)
1tyr-01,1tyr-01-s,1tyr-02,1tyr-02-s,1tyr-03,1tyr-03-s,1tyr-04,1tyr-04-s,1tyr-05,1tyr-05-s,1tyr-06,1tyr-07,1tyr-07-s,1tyr-08,1tyr-08-s,1tyr-09,1tyr-09-s,1tyr-10,1tyr-10-s	1tyr(TTR)	anti-inflammatory	(75)
2dq7-01,2dq7-02,2dq7-02-s,2dq7-03,2dq7-03-s,2dq7-04,2dq7-05,2dq7-05-s,2dq7-06,2dq7-06-s,2dq7-07,2dq7-07-s,2dq7-08,2dq7-09,2dq7-09-s,2dq7-10	2dq7(FYN)	anti-SARS-CoV-2 anti-inflammatory	(76)
3cqu-01,3cqu-01-s,3cqu-02,3cqu-02-s,3cqu-03,3cqu-03-s,3cqu-04,3cqu-04-s,3cqu-05,3cqu-05-s,3cqu-06,3cqu-06-s,3cqu-07,3cqu-07-s,3cqu-08,3cqu-08-s,3cqu-09,3cqu-09-s,3cqu-10,3cqu-10-s	3cqu(AKT1)	anti-SARS-CoV-2 anti-inflammatory	(80)
3cqw-01,3cqw-01-s,3cqw-02,3cqw-03, 3cqw-04, 3cqw-04-s, 3cqw-05, 3cqw-05-s, 3cqw-06, 3cqw-06-s, 3cqw-07, 3cqw-07-s, 3cqw-08, 3cqw-09, 3cqw-10, 3cqw-10-s	3cqw(AKT1)	anti-SARS-CoV-2 anti-inflammatory	(80)
2orj-01,2orj-02,2orj-03,2orj-04	2orj(SFTPD)	anti-SARS-CoV-2 anti-inflammatory	(77)
2x0u-01,2x0u-01-s	2x0u(TP53)	anti-SARS-CoV-2 anti-inflammatory	(78)
2x0v-01,2x0v-01-s	2x0v(TP53)	anti-SARS-CoV-2 anti-inflammatory	(78)
3beg-01,3beg-02	3beg(SRPK1)	anti-SARS-CoV-2	(79)

### 3.5 Construction of protein-protein interaction network

**Figure 3:**
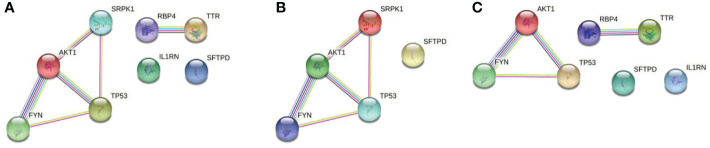
**(A)** PPI network of 8 potential targets. **(B)** PPI network of the SARS-CoV-2 specific targets. **(C)** PPI network of the inflammatory targets. Associations are meant to be specific and meaningful, i.e. proteins jointly contribute to a shared function; this does not necessarily mean they are physically binding to each other. The light-blue edges denote known interactions from curated databases. The pink edges show that the known interactions were determined by experimental methods. The yellow edges demonstrate that the predicted interactions arose from text-mining. The black edges denote that the predicted interactions arose from co-expression. The lavender edges show that the predicted interactions arose from protein homology. The dark-blue edges denote that the predicted interactions arose from gene co-occurrence.

PPI network was constructed by String with the potential targets of the 62 active components in QFPD. It can be seen from **Figure 3A** that the network contained 8 nodes and 6 edges. The nodes represented the targets, and the edges represented the interactions between the targets. The more edges the node had, the more critical it was in the network. TP53 and AKT1, with a high degree of connectivity, were core genes that may play an essential role in treating COVID-19 with QFPD. The network in **Figure 3B** contained 5 nodes and 5 edges. If medium confidence≧0.4 was selected as the screening criteria, the candidate targets were TP53, AKT1, FYN, and SRPK1. If the highest confidence≧0.9 was selected as the screening criteria, the candidate targets were TP53, AKT1. The network in **Figure 3C** contained 7 nodes and 4 edges. If medium confidence≧0.4 was selected as the screening criteria, the candidate targets were TP53, AKT1, RBP4, TTR, and FYN. If the highest confidence≧0.9 was selected as the screening criteria, the candidate targets were TP53, AKT1, RBP4, and TTR.

### 3.6 Construction of “herb-active component-target” interaction network diagram

**Figure 4:**
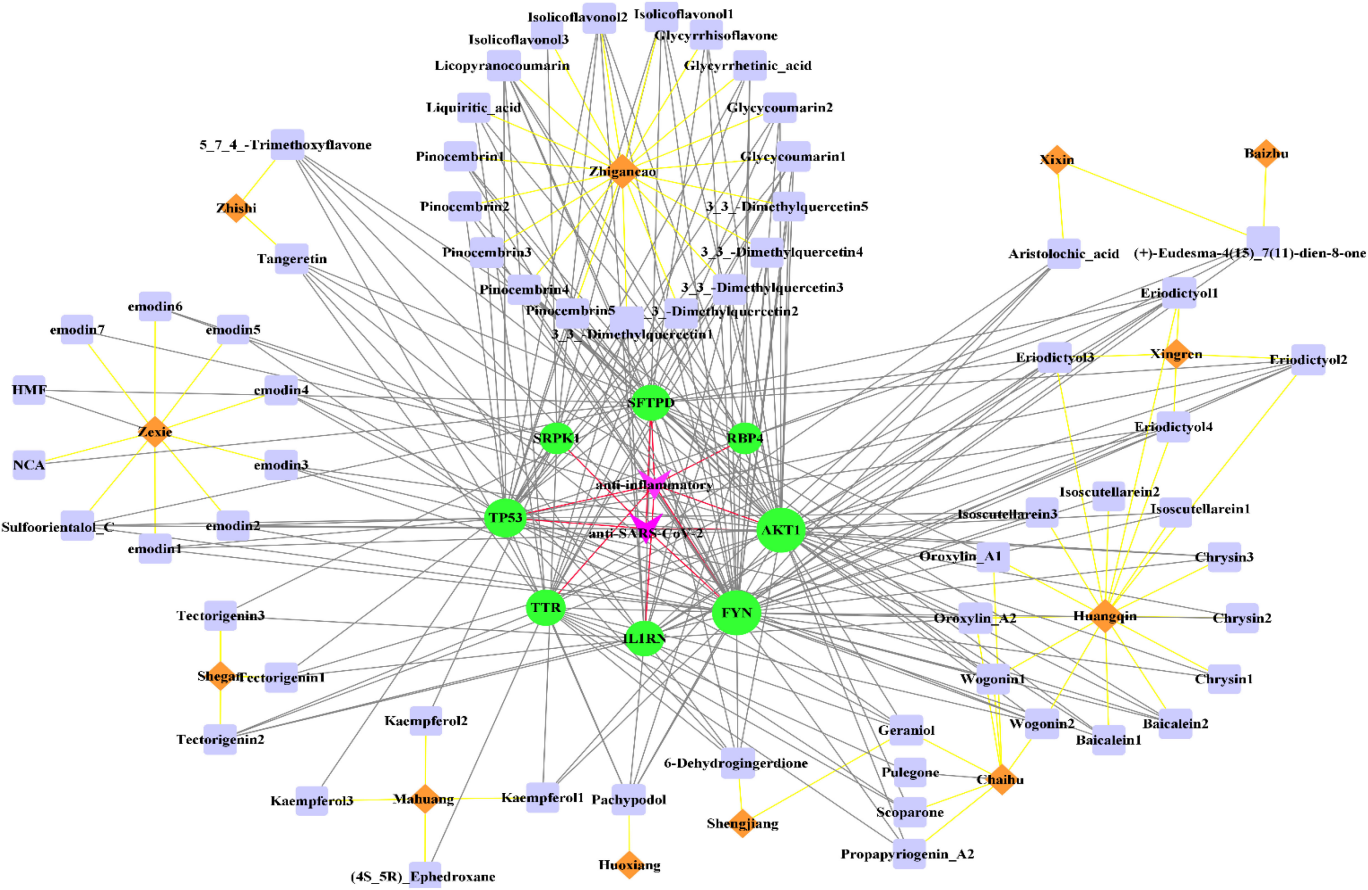
The “herb-active component-target” interaction network diagram for the treatment of COVID-19. 12 herbs (Mahuang, Zhishi, Huoxiang, Zexie, Shegan, Shengjiang, Chaihu, Huangqin, Xingren, Baizhu, Xixin, Zhigancao) in QFPD are marked in orange; 62 active components screened from 12 herbs with anti-SARS-CoV-2 or anti-inflammatory effects are marked in purple; 8 potential targets of QFPD are marked in green, and the properties of the targets are marked in pink. The black line represents that a certain active component comes from a certain herb; the yellow line represents the interaction between the active component and the target, and the red line represents a certain target is against inflammation or SARS-CoV-2.

As shown in **Figure 4**, 62 active components from 12 herbs (Mahuang, Zhishi, Huoxiang, Zexie, Shegan, Shengjiang, Chaihu, Huangqin, Xingren, Baizhu, Xixin, and Zhigancao) have anti-SARS-CoV-2 or anti-inflammatory effects. Among them, Eriodictyol was common in Xixin and Huangqin. Wogonin was common in Chaihu and Huangqin. Geraniol was common in Chaihu and Shengjiang. (+)-Eudesma-4(15),7(11)-dien-8-one was common in Xixin and Baizhu. It can be seen from **Table 3** that, Kaempferol2, Kaempferol3, Emodin7, and Isolicoflavonol3 only had anti-SARS-CoV-2 effects, (4S_5R) Ephedroxane and Pulegone only had anti-inflammatory effects. The other 56 components were both anti-inflammatory and anti-SARS-CoV-2. Among these components, Quercetin, Wogonin, and Emodin were able to interfere with various stages of the coronavirus entry and replication cycle (59, 81, 82). Kaempferol could be used as an antiviral drug against the 3a Channel Protein of Coronavirus (55). Baicalein had a high affinity for SARS-CoV-2 3CLpro (83). They were identified as candidate active components for COVID-19. In addition, each target was bound to two or more active components, indicating that these targets could be affected by multiple active components simultaneously to exert different effects.

**Table 3:** 62 components determined by multiple computational processes in QFPD.

Active components	Effect
Kaempferol3	anti-SARS-CoV-2
Emodin7	anti-SARS-CoV-2
Kaempferol2	anti-SARS-CoV-2
Isolicoflavonol3	anti-SARS-CoV-2
(4S_5R)_Ephedroxane	anti-inflammatory
Pulegone	anti-inflammatory
Pachypodol	anti-SARS-CoV-2, anti-inflammatory
3_3_-Dimethylquercetin1	anti-SARS-CoV-2, anti-inflammatory
Glycyrrhisoflavone	anti-SARS-CoV-2, anti-inflammatory
Oroxylin_A2	anti-SARS-CoV-2, anti-inflammatory
Wogonin1	anti-SARS-CoV-2, anti-inflammatory
Isolicoflavonol2	anti-SARS-CoV-2, anti-inflammatory
6-Dehydrogingerdione	anti-SARS-CoV-2, anti-inflammatory
Isolicoflavonol1	anti-SARS-CoV-2, anti-inflammatory
3_3_-Dimethylquercetin3	anti-SARS-CoV-2, anti-inflammatory
Baicalein2	anti-SARS-CoV-2, anti-inflammatory
3_3_-Dimethylquercetin4	anti-SARS-CoV-2, anti-inflammatory
Emodin3	anti-SARS-CoV-2, anti-inflammatory
Emodin1	anti-SARS-CoV-2, anti-inflammatory
Wogonin2	anti-SARS-CoV-2, anti-inflammatory
Pinocembrin3	anti-SARS-CoV-2, anti-inflammatory
Glycycoumarin2	anti-SARS-CoV-2, anti-inflammatory
3_3_-Dimethylquercetin2	anti-SARS-CoV-2, anti-inflammatory
Eriodictyol2	anti-SARS-CoV-2, anti-inflammatory
Tangeretin	anti-SARS-CoV-2 anti-inflammatory
Aristolochic_acid	anti-SARS-CoV-2, anti-inflammatory
(+)-Eudesma-4(15)_7(11)-dien-8-one	anti-SARS-CoV-2, anti-inflammatory
Glycycoumarin1	anti-SARS-CoV-2, anti-inflammatory
5_7_4_-Trimethoxyflavone	anti-SARS-CoV-2, anti-inflammatory
Glycyrrhetinic_acid	anti-SARS-CoV-2, anti-inflammatory
Geraniol	anti-SARS-CoV-2, anti-inflammatory
Licopyranocoumarin	anti-SARS-CoV-2, anti-inflammatory
Propapyriogenin_A2	anti-SARS-CoV-2, anti-inflammatory
Pinocembrin2	anti-SARS-CoV-2, anti-inflammatory
Isoscutellarein3	anti-SARS-CoV-2, anti-inflammatory
Liquiritic_acid	anti-SARS-CoV-2, anti-inflammatory
Sulfoorientalol_C	anti-SARS-CoV-2 anti-inflammatory
Eriodictyol1	anti-SARS-CoV-2, anti-inflammatory
Emodin2	anti-SARS-CoV-2, anti-inflammatory
Tectorigenin2	anti-SARS-CoV-2, anti-inflammatory
Emodin4	anti-SARS-CoV-2, anti-inflammatory
Pinocembrin5	anti-SARS-CoV-2, anti-inflammatory
Chrysin3	anti-SARS-CoV-2, anti-inflammatory
Eriodictyol4	anti-SARS-CoV-2, anti-inflammatory
Baicalein1	anti-SARS-CoV-2, anti-inflammatory
Isoscutellarein1	anti-SARS-CoV-2, anti-inflammatory
Isoscutellarein2	anti-SARS-CoV-2, anti-inflammatory
Eriodictyol3	anti-SARS-CoV-2, anti-inflammatory
Kaempferol1	anti-SARS-CoV-2 anti-inflammatory
Chrysin1	anti-SARS-CoV-2, anti-inflammatory
Chrysin2	anti-SARS-CoV-2, anti-inflammatory
Pinocembrin1	anti-SARS-CoV-2, anti-inflammatory
Pinocembrin4	anti-SARS-CoV-2, anti-inflammatory
3_3_-Dimethylquercetin5	anti-SARS-CoV-2, anti-inflammatory
HMF	anti-SARS-CoV-2, anti-inflammatory
Oroxylin_A1	anti-SARS-CoV-2, anti-inflammatory
Tectorigenin3	anti-SARS-CoV-2, anti-inflammatory
Tectorigenin1	anti-SARS-CoV-2, anti-inflammatory
NCA	anti-SARS-CoV-2, anti-inflammatory
Emodin5	anti-SARS-CoV-2, anti-inflammatory
Emodin6	anti-SARS-CoV-2, anti-inflammatory
Scoparone	anti-SARS-CoV-2, anti-inflammatory

### 3.7 Pathway analysis of QFPD

**Figure 5:**
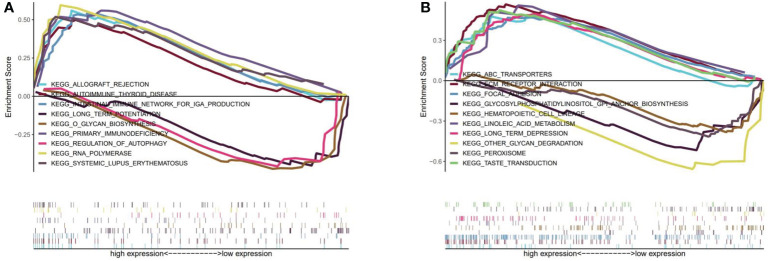
**(A)** GSEA analysis for TP53. **(B)** GSEA analysis for AKT1.

We identified TP53 and AKT1 as core targets based on the PPI network. Therefore, according to the expression of TP53 or AKT1, we divided them into high and low expression groups for GSEA analysis. P-value<0.05 was considered statistically significant. **Figure 5A** showed that 6 significant pathways were enriched in the TP53 high expression group: RNA polymerase, primary immunodeficiency, intestinal immune network for IGA production, systemic lupus erythematosus, allograft rejection, and autoimmune thyroid disease. 3 pathways were enriched in the TP53 low expression group: O glycan biosynthesis, regulation of autophagy, and long-term potentiation. 20 meaningful pathways were enriched in the AKT1 high expression group. We showed the first six significant enrichments in **Figure 5B**, which were taste transduction, ECM receptor interaction, focal adhesion, ABC transporters, long term depression, and linoleic acid metabolism. 3 pathways were enriched in the AKT1 low expression group, namely other glycan degradation, glycosylphosphatidylinositol GPI anchor biosynthesis, peroxisome, and hematopoietic cell lineage.

**Figure 6:**
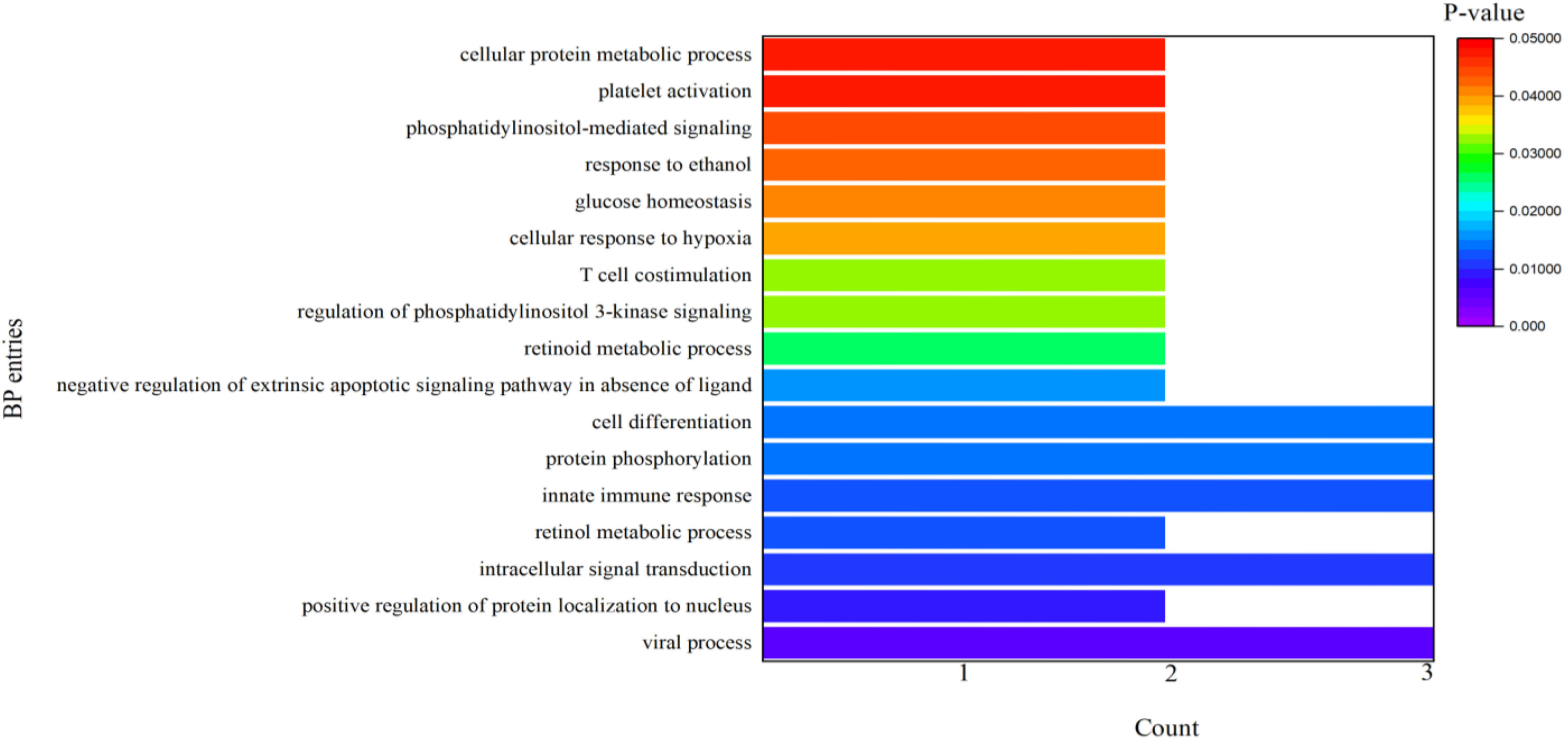
BP entries for the potential targets of active components in QFPD.

**Figure 7:**
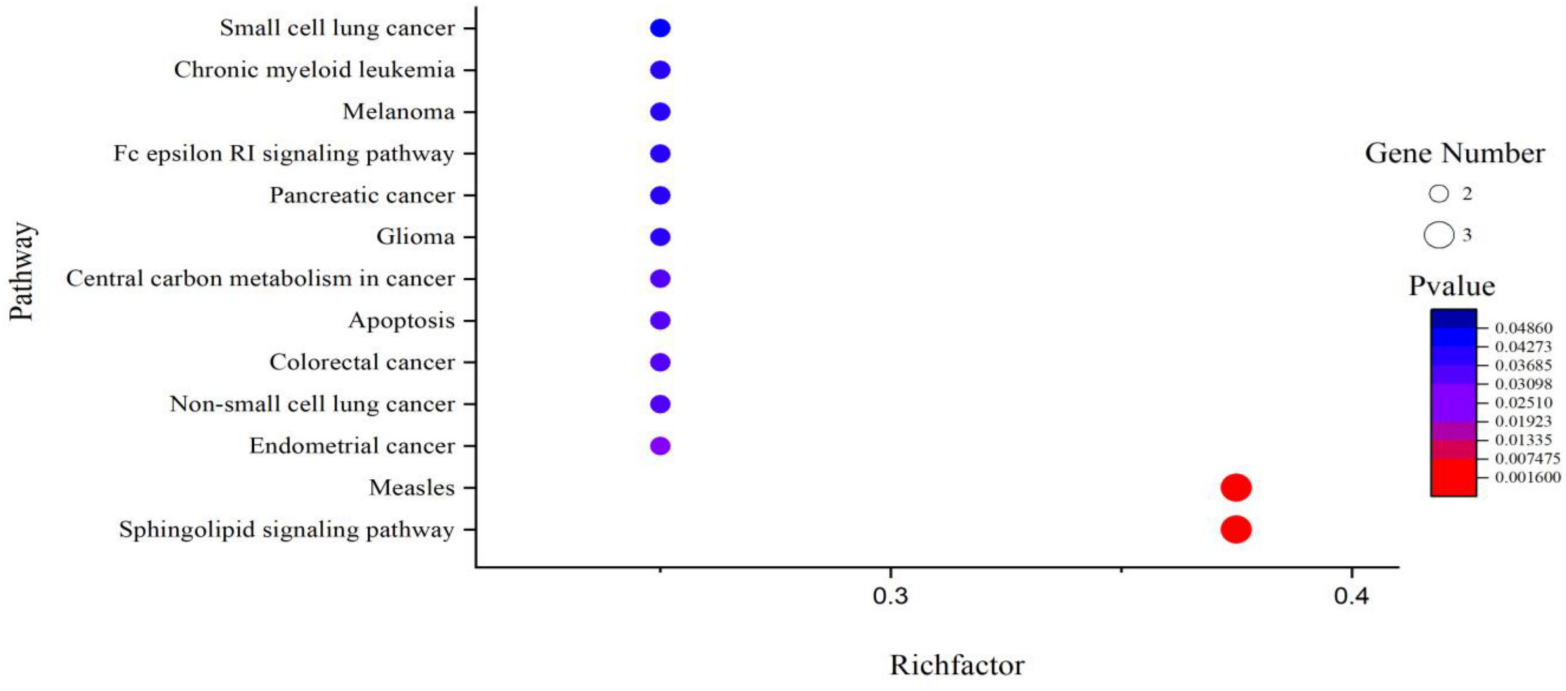
KEGG pathway analysis for the SARS-CoV-2 specific targets of active components in QFPD. The rich factor indicates the ratio of the number of target genes belonging to a pathway to the total number of annotated target genes in the pathway. The higher value for this ratio corresponds to a higher level of enrichment. The size and color of the bubble indicates the number and significant characters of target genes that enriched in pathways.

It can be seen from **Table 4** that 8 potential targets of QFPD (RBP4, IL1RN, TTR, FYN, SFTPD, TP53, SRPK1, and AKT1) were mainly enriched in 21 BP entries. 17 BP entries were determined with P-value<0.05 (**Figure 6**). The core BP entries mainly included the viral process, intracellular signal transduction, innate immune response, protein phosphorylation, and cell differentiation. During SARS-CoV-2 infection, the innate immune system experienced substantial disturbance (84). Several of the cytokines involved in the reaction employ a distinct intracellular signaling pathway mediated by Janus kinases (85). Researchers studied the perturbation in protein phosphorylation during SARS-CoV-2 infection by mass spectrometry, and the results showed that large changes were observed in protein phosphorylation (86). This evidences verified that QFPD regulates diseases through a variety of biological processes. The core MF entries mainly included protein binding, identical protein binding, enzyme binding, and ATP binding. A recent study reported that SARS-CoV-2 enters the host cells through the binding of its spike protein to the cell surface-expressing angiotensin-converting enzyme 2 (ACE2) (87). Therefore, inhibiting the binding of some specific proteins or enzymes may attenuate the progression of COVID-19. The core CC entries mainly included protein complex and extracellular space. The GO analysis results showed that AKT1, FYN, TP53, TTR, and RBP4 were key targets involved in regulation. The KEGG analysis results showed that AKT1, FYN, and TP53 were key targets involved in regulation (**Table 5**). The top 13 KEGG pathways are shown in **Figure 7**. The core pathways mainly included Sphingolipid signaling pathway, Fc epsilon RI signaling pathway, Apoptosis, and Measles. Sphingolipids play a vital role in protecting the lung from damages (88). The Fc epsilon RI signaling pathway is associated with cytokine secretion and inflammatory responses (89). Based on previous data, SARS-CoV-2 may have the ability to induce endogenous and exogenous apoptotic pathways and stimulate T cell apoptosis (90). Therefore, we speculate that the active components in QFPD may play an important role in the therapeutic of COVID-19 by multiple pathways.

**Table 4:** BP, MF and CC entries of GO analysis.

	Item	Count	P-value	Genes
BP	viral process	3	0.0063	FYN, TP53, SRPK1
	intracellular signal transduction	3	0.0111	AKT1, FYN, SRPK1
	innate immune response	3	0.0126	SFTPD, FYN, SRPK1
	protein phosphorylation	3	0.0141	AKT1, FYN, SRPK1
	cell differentiation	3	0.0145	AKT1, FYN, TP53
	positive regulation of protein localization to nucleus	2	0.0087	AKT1, FYN
	retinol metabolic process	2	0.0124	RBP4, TTR
	negative regulation of extrinsic apoptotic signaling pathway in absence of ligand	2	0.0153	AKT1, FYN
	retinoid metabolic process	2	0.0252	RBP4, TTR
	regulation of phosphatidylinositol 3-kinase signaling	2	0.0321	AKT1, FYN
	T cell costimulation	2	0.0321	AKT1, FYN
	cellular response to hypoxia	2	0.0393	AKT1, TP53
	glucose homeostasis	2	0.0414	RBP4, AKT1
	response to ethanol	2	0.0430	RBP4, FYN
	phosphatidylinositol-mediated signaling	2	0.0434	AKT1, FYN
	platelet activation	2	0.0470	AKT1, FYN
	cellular protein metabolic process	2	0.0482	TTR, SFTPD
	regulation of signal transduction by p53 class mediator	2	0.0506	AKT1, TP53
	negative regulation of gene expression	2	0.0557	AKT1, FYN
	cellular response to DNA damage stimulus	2	0.0836	AKT1, TP53
	regulation of apoptotic process	2	0.0855	FYN, TP53
MF	protein binding	8	0.0103	IL1RN, RBP4, TTR, SFTPD, AKT1, FYN, TP53, SRPK1
	identical protein binding	4	0.0027	TTR, AKT1, FYN, TP53
	ATP binding	4	0.0185	AKT1, FYN, TP53, SRPK1
	enzyme binding	3	0.0076	AKT1, FYN, TP53
	protein heterodimerization activity	3	0.0145	RBP4, TTR, TP53
	protein phosphatase 2A binding	2	0.0111	AKT1, TP53
CC	protein complex	4	0.0004	RBP4, TTR, AKT1, TP53
	extracellular space	4	0.0112	IL1RN, RBP4, TTR, SFTPD
	mitochondrion	3	0.0875	AKT1, FYN, TP53
	nuclear matrix	2	0.0367	TP53, SRPK1

**Table 5:** 13 KEGG pathways.

Pathway	Count	P-value	Genes
Sphingolipid signaling pathway	3	0.0018	AKT1, FYN, TP53
Measles	3	0.0022	AKT1, FYN, TP53
Endometrial cancer	2	0.0299	AKT1, TP53
Non-small cell lung cancer	2	0.0322	AKT1, TP53
Colorectal cancer	2	0.0356	AKT1, TP53
Apoptosis	2	0.0356	AKT1, TP53
Central carbon metabolism in cancer	2	0.0367	AKT1, TP53
Glioma	2	0.0373	AKT1, TP53
Pancreatic cancer	2	0.0373	AKT1, TP53
Fc epsilon RI signaling pathway	2	0.0390	AKT1, FYN
Melanoma	2	0.0407	AKT1, TP53
Chronic myeloid leukemia	2	0.0412	AKT1, TP53
Small cell lung cancer	2	0.0485	AKT1, TP53

## Conclusion

In this study, we used various network pharmacology methods combined with CADD techniques to reveal the diversity of potential targets and therapeutic pathways for QFPD against COVID-19. We found that RBP4, IL1RN, TTR, FYN, SFTPD, TP53, SRPK1, and AKT1 are highly related to COVID-19. QFPD could act on multiple pathways, including viral process, immunodeficiency, RNA polymerase, Sphingolipid signaling pathway, and taste transduction. The results showed that QFPD has “multi-component, multi-target, and multi-pathway” characteristics in regulating inflammation, viral infection, cellular damage, and immune responses. Our work helps to establish the basic theory of TCM for the treatment of COVID-19.

## Data availability statement

The original contributions presented in the study are included in the article/**Supplementary Material**. Further inquiries can be directed to the corresponding author.

## Author contributions

ZW conducted the literature review, designed the research system, collated data information, and wrote the manuscript. JZ contributed to the manuscript's revision and guided the calculation procee. HG contributed to the conception and design of the study. All authors participate in the revision, reading, and approval the submitted version.
