# Supplementary material for: Computer-aided drug design combined network pharmacology to explore anti-SARS-CoV-2 or anti-inflammatory targets and mechanisms of Qingfei Paidu Decoction for COVID-19
Source: Front Immunol. 2022 Dec 23;13:1015271. doi: 10.3389/fimmu.2022.1015271 (PMC9816407; doi:10.3389/fimmu.2022.1015271)
Supplement: Supplementary file 1 [file DataSheet_1.pdf]

Figure captions

Figure S1. The structures of 64 active components.

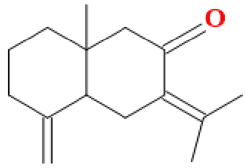

(+)-Eudesma-4(15)_7(11)-dien-8-one

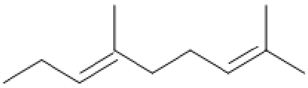

Geraniol

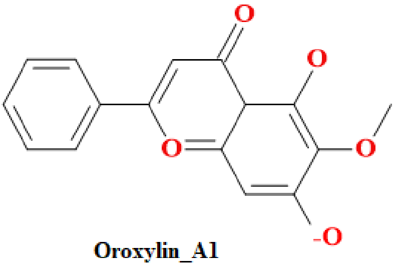

Oroxylin_A1

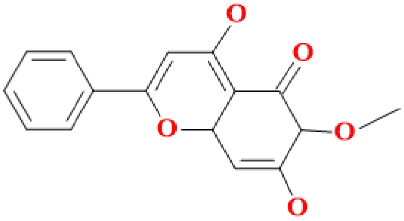

Oroxylin_A2

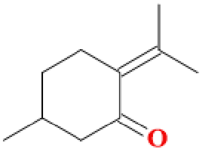

Pulegone

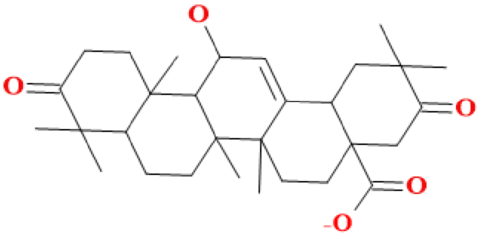

Propapyriogenin_A2

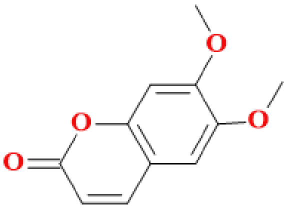

Scoparone

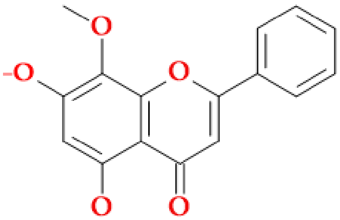

Wogonin1

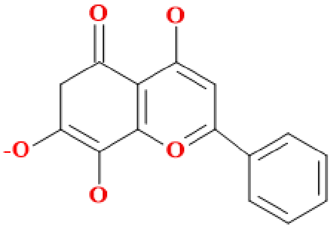

Wogonin2

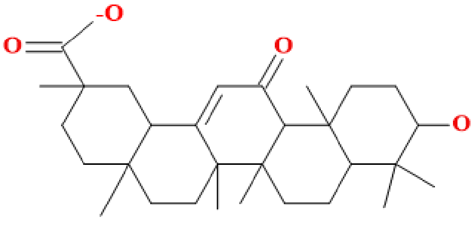

Liquiritic_acid

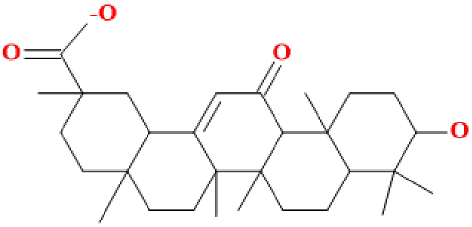

Glycyrrhetinic_acid

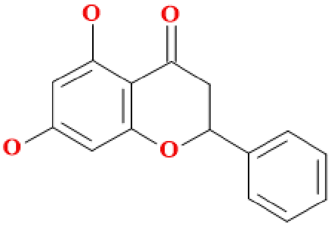

Pinocembrin1

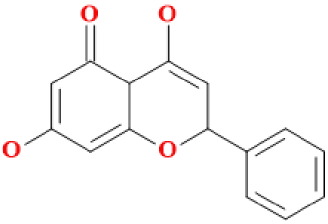

Pinocembrin2

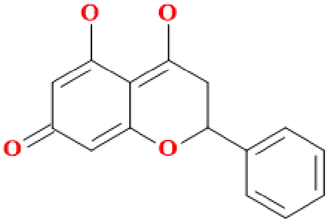

Pinocembrin3

Figure S1. The structures of 64 active components.

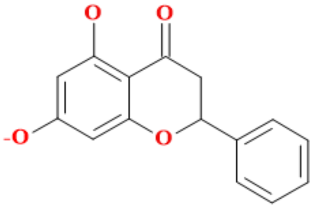
Pinocembrin4

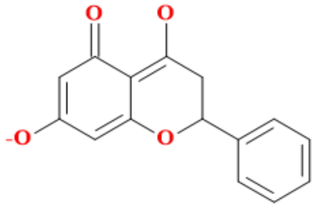
Pinocembrin5

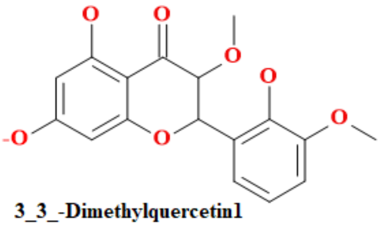
3_3_-Dimethylquercetin1

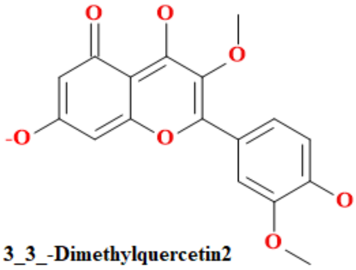
3_3_-Dimethylquercetin2

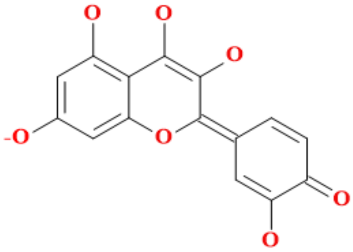
3_3_-Dimethylquercetin3

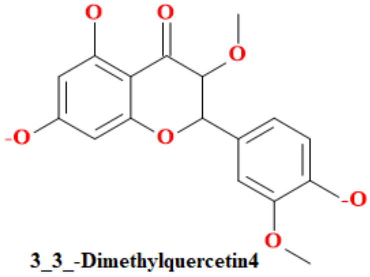
3_3_-Dimethylquercetin4

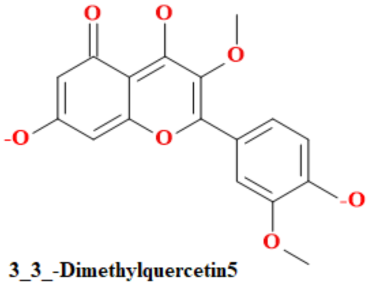
3_3_-Dimethylquercetin5

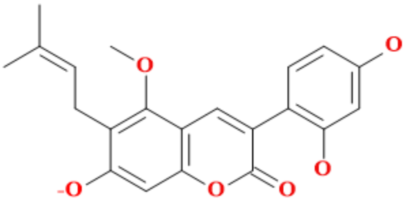
Glycycoumarin1

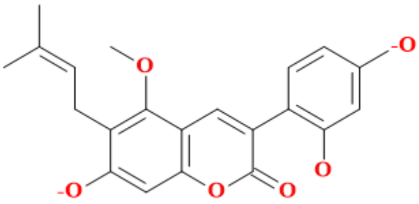
Glycycoumarin2

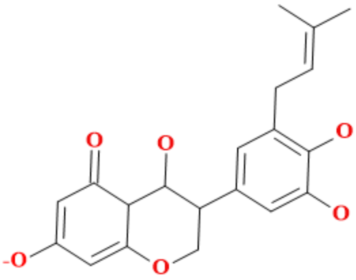
Glycyrrhisoflavone

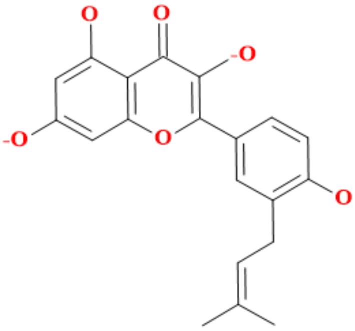
Isolicoflavonol1

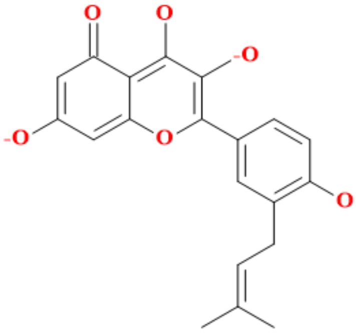
Isolicoflavonol2

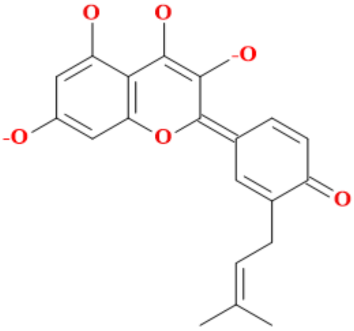
Isolicoflavonol3

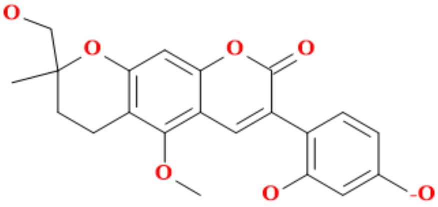
Licopyranocoumarin

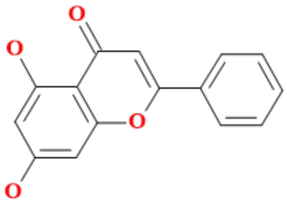
Chrysin1

Figure S1 continued. The structures of 64 active components.

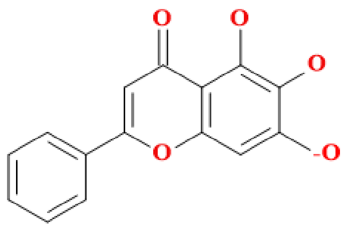

Baicalein1

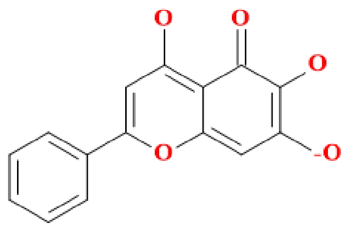

Baicalein2

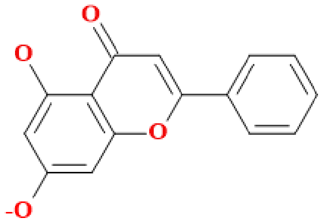

Chrysin2

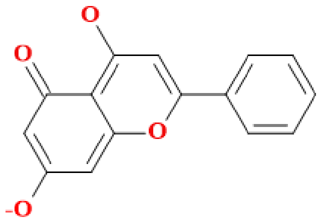

Chrysin3

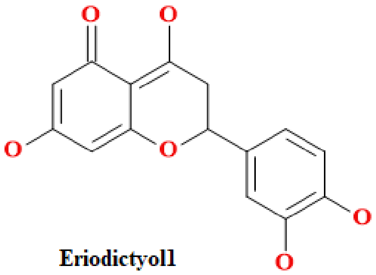

Eriodictyol1

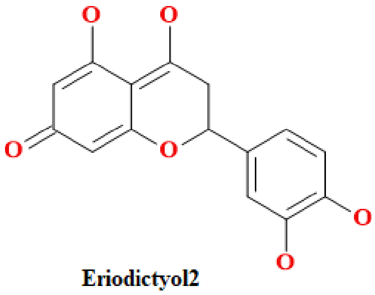

Eriodictyol2

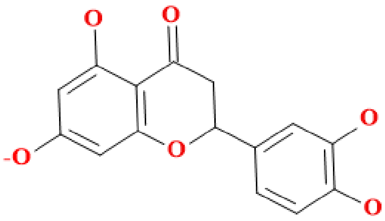

Eriodictyol3

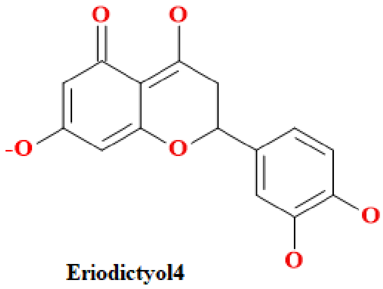

Eriodictyol4

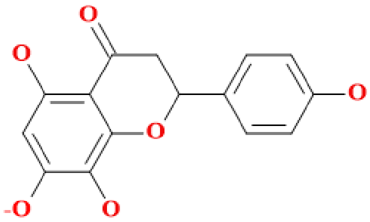

Isoscutellarein1

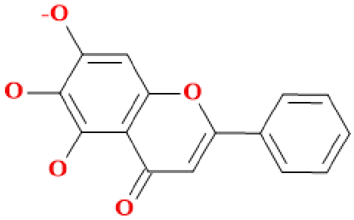

Isoscutellarein2

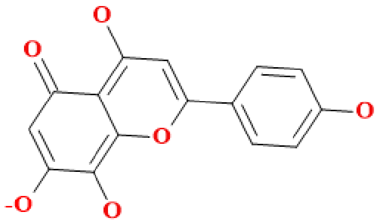

Isoscutellarein3

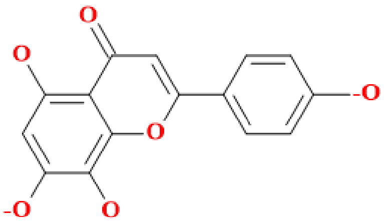

Isoscutellarein4

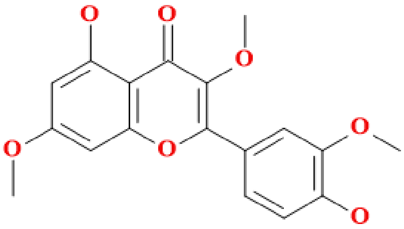

Pachypodol

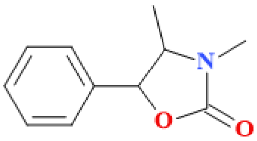

(4S_5R)_Ephedroxane

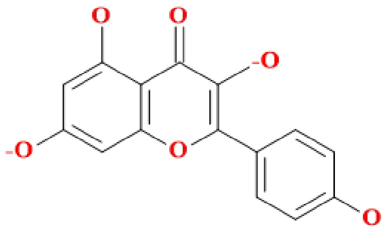

Kaempferol1

Figure S1 continued. The structures of 64 active components.

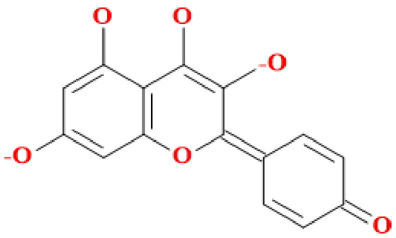

Kaempferol2

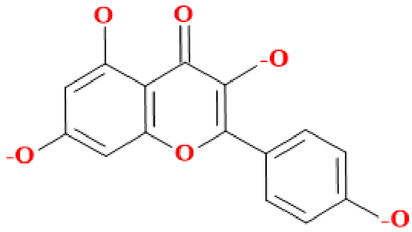

Kaempferol3

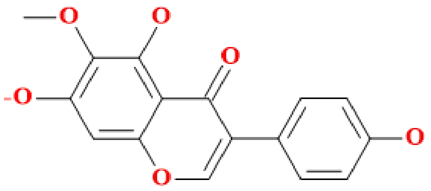

Tectorigenin1

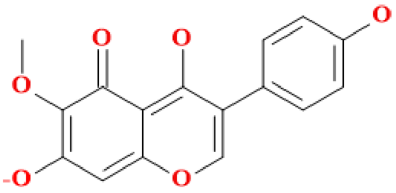

Tectorigenin2

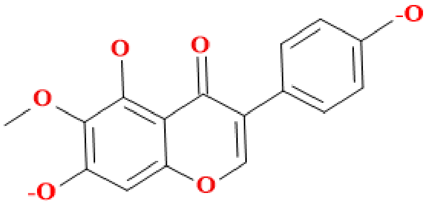

Tectorigenin3

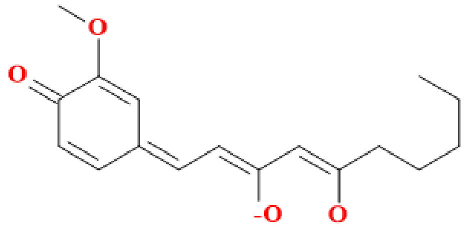

6-Dehydrogingerdione

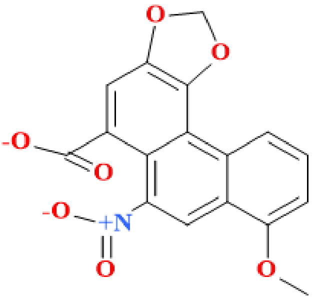

Aristolochic_acid

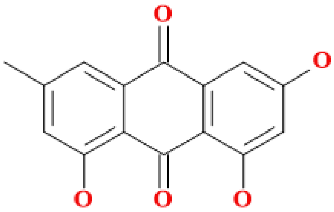

emodin1

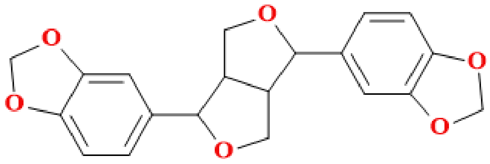

Sesamin

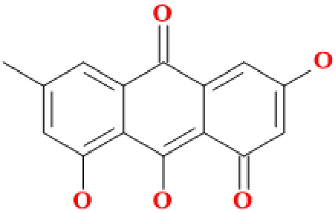

emodin2

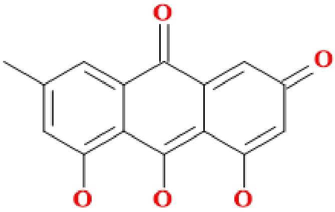

emodin3

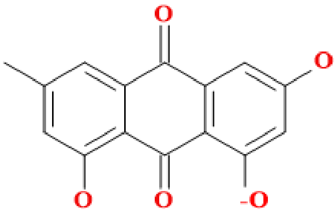

emodin4

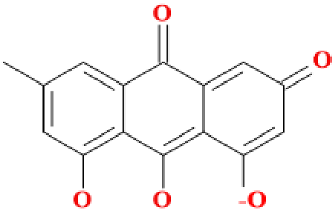

emodin5

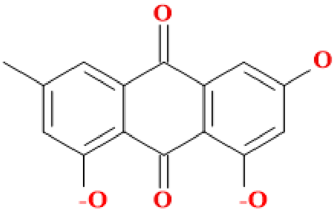

emodin6

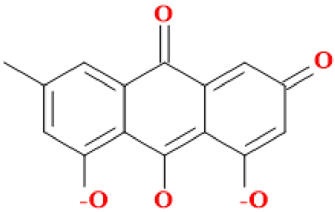

emodin7

Figure S1 continued. The structures of 64 active components.

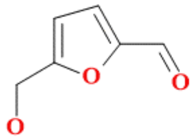

HMF

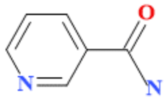

NCA

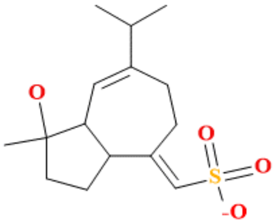

Sulfoorientalol_C

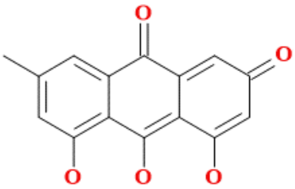

Tangeretin

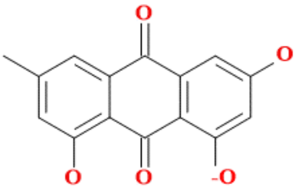

5_7_4_-Trimethoxyflavone

Figure S1 continued. The structures of 64 active components.
