# Supplementary material for: Computer-aided drug design combined network pharmacology to explore anti-SARS-CoV-2 or anti-inflammatory targets and mechanisms of Qingfei Paidu Decoction for COVID-19
Source: Front Immunol. 2022 Dec 23;13:1015271. doi: 10.3389/fimmu.2022.1015271 (PMC9816407; doi:10.3389/fimmu.2022.1015271)
Supplement: Supplementary file 2 [file Table_1.docx]

**Table S1** TOPKAT results of the most promising active components.

| Active components name | Rodent Carcinogenicity | Ames | Rat Oral LD50 | Aerobic Biodegradability |
| --- | --- | --- | --- | --- |
| HMF | Non-Carcinogen | 0.757822 | 2.4488 | 0.75949 |
| NCA | Non-Carcinogen | 0.658514 | 1.01744 | 0.50199 |
| Emodin1 | Non-Carcinogen | 0.940641 | 3.14451 | 0.123498 |
| Emodin2 | Non-Carcinogen | 0.649375 | 1.90911 | 0.289369 |
| Emodin3 | Non-Carcinogen | 0.62737 | 1.90911 | 0.309194 |
| Emodin4 | Non-Carcinogen | 0.789781 | 2.01525 | 0.158668 |
| Emodin5 | Non-Carcinogen | 0.637511 | 1.20919 | 0.377272 |
| Emodin3 | Non-Carcinogen | 0.74962 | 1.11315 | 0.227532 |
| Emodin7 | Non-Carcinogen | 0.639872 | 0.675824 | 0.430553 |
| Sesamin | Non-Carcinogen | 0.535598 | 0.489369 | 0.609635 |
| Tangeretin | Non-Carcinogen | 0.305403 | 0.300664 | 0.508804 |
| Eriodictyol1 | Non-Carcinogen | 0.550327 | 1.12343 | 0.552479 |
| Eriodictyol2 | Non-Carcinogen | 0.51162 | 1.11873 | 0.57644 |
| Eriodictyol3 | Non-Carcinogen | 0.072989 | 1.04107 | 0.493795 |
| Eriodictyol4 | Non-Carcinogen | 0.538651 | 0.711718 | 0.545518 |
| stearic_acid | Non-Carcinogen | 0.011093 | 17.2093 | 0.912716 |
| Pachypodol | Non-Carcinogen | 0.669673 | 1.14754 | 0.481777 |
| Kaempferol1 | Non-Carcinogen | 0.659298 | 0.38789 | 0.418633 |
| Kaempferol2 | Non-Carcinogen | 0.586853 | 0.136067 | 0.43831 |
| Kaempferol3 | Non-Carcinogen | 0.677311 | 0.14359 | 0.401749 |
| Tectorigenin1 | Non-Carcinogen | 0.148444 | 0.676274 | 0.494654 |
| Tectorigenin2 | Non-Carcinogen | 0.524173 | 0.321101 | 0.589578 |
| Tectorigenin3 | Non-Carcinogen | 0.252644 | 0.231626 | 0.51461 |
| Isoscutellarein1 | Non-Carcinogen | 0.292071 | 0.509274 | 0.384727 |
| Isoscutellarein2 | Non-Carcinogen | 0.623944 | 0.215693 | 0.395562 |
| Isoscutellarein3 | Non-Carcinogen | 0.629102 | 0.228494 | 0.446789 |
| Isoscutellarein4 | Non-Carcinogen | 0.407423 | 0.2017 | 0.443037 |
| Cinnamaldehyde | Non-Carcinogen | 0.693324 | 1.30852 | 0.512909 |
| Aristolochic_acid | Non-Carcinogen | 0.982165 | 0.334281 | 0.19355 |
| Sulfoorientalol_C | Non-Carcinogen | 0.316246 | 0.565963 | 0.709589 |
| N-Methylephedrine | Non-Carcinogen | 0.6402 | 3.11744 | 0.533832 |
| 6-Dehydrogingerdione | Non-Carcinogen | 0.303769 | 0.664528 | 0.814471 |
| (4S_5R)_Ephedroxane | Non-Carcinogen | 0.668916 | 0.991625 | 0.562176 |
| 5_7_4_-Trimethoxyflavone | Non-Carcinogen | 0.208092 | 0.359721 | 0.538843 |

**Table S1** **continued** TOPKAT results of the most promising active components.

| Active components name | Rodent Carcinogenicity | Ames | Rat Oral LD50 | | Aerobic Biodegradability |
| --- | --- | --- | --- | --- | --- |
| Geraniol | Non-Carcinogen | 0.065418 | 3.66384 | | 0.923213 |
| Pulegone | Non-Carcinogen | 0.198852 | 1.65598 | | 0.722582 |
| Scoparone | Non-Carcinogen | 0.636048 | 1.14097 | | 0.7564 |
| Chrysin | Non-Carcinogen | 0.111863 | 0.258015 | | 0.417042 |
| Baicalein1 | Non-Carcinogen | 0.205017 | 0.172307 | | 0.436287 |
| Baicalein2 | Non-Carcinogen | 0.654725 | 0.149751 | | 0.515193 |
| Chrysin1 | Non-Carcinogen | 0.174942 | 0.142926 | | 0.423573 |
| Chrysin2 | Non-Carcinogen | 0.66754 | 0.122014 | | 0.535654 |
| Wogonin1 | Non-Carcinogen | 0.351008 | 0.0938626 | | 0.447535 |
| Wogonin2 | Non-Carcinogen | 0.635343 | 0.0992736 | | 0.581676 |
| Oroxylin_A1 | Non-Carcinogen | 0.184036 | 0.0913893 | | 0.463336 |
| Oroxylin_A2 | Non-Carcinogen | 0.627359 | 0.0992553 | | 0.581676 |
| Liquiritic_acid | Non-Carcinogen | 8.87E-06 | 1.27097 | | 0.730006 |
| Pinocembrin1 | Non-Carcinogen | 0.077954 | 0.544404 | | 0.535875 |
| Pinocembrin2 | Non-Carcinogen | 0.627138 | 0.419365 | | 0.631557 |
| Pinocembrin3 | Non-Carcinogen | 0.594698 | 0.41769 | | 0.654128 |
| Pinocembrin4 | Non-Carcinogen | 0.167847 | 0.301578 | | 0.542622 |
| Pinocembrin5 | Non-Carcinogen | 0.621283 | | 0.229593 | 0.644483 |
| Isolicoflavonol1 | Non-Carcinogen | 0.588819 | | 0.368119 | 0.440223 |
| Isolicoflavonol2 | Non-Carcinogen | 0.570791 | | 0.29753 | 0.548377 |
| Isolicoflavonol3 | Non-Carcinogen | 0.352212 | | 0.195225 | 0.620353 |
| Glycycoumarin4 | Non-Carcinogen | 0.438414 | | 0.529674 | 0.642388 |
| Glycycoumarin5 | Non-Carcinogen | 0.486625 | | 0.183659 | 0.623026 |
| Glycyrrhisoflavone | Non-Carcinogen | 0.524591 | | 0.485387 | 0.608741 |
| Licopyranocoumarin | Non-Carcinogen | 0.34799 | | 0.375377 | 0.510166 |
| Propapyriogenin_A2 | Non-Carcinogen | 0.011915 | | 1.75917 | 0.731646 |
| Glycyrrhetinic_acid | Non-Carcinogen | 8.87E-06 | | 1.27097 | 0.730006 |
| 3_3_-Dimethylquercetin1 | Non-Carcinogen | 0.631479 | | 0.528344 | 0.518521 |
| 3_3_-Dimethylquercetin2 | Non-Carcinogen | 0.550035 | | 0.636029 | 0.622739 |
| 3_3_-Dimethylquercetin3 | Non-Carcinogen | 0.55875 | | 0.218344 | 0.493588 |
| 3_3_-Dimethylquercetin4 | Non-Carcinogen | 0.688634 | | 0.180996 | 0.502659 |
| 3_3_-Dimethylquercetin5 | Non-Carcinogen | 0.604492 | | 0.21979 | 0.609962 |
| (+)-Eudesma-4(15)_7(11)-dien-8-one | Non-Carcinogen | 0.122225 | | 2.38961 | 0.724887 |
| 1-(3_4_5-Trimethoxyphenyl)-2-propenyl_2-(2-methyl-2Z-butenoyloxymethyl)-2Z-butenoate | Non-Carcinogen | 0.389345 | | 2.11348 | 0.746216 |
